# Comprehensive geriatric assessment as a useful tool in predicting adverse events in elderly patients with diffuse large B-cell lymphoma

**DOI:** 10.1038/s41598-022-07164-w

**Published:** 2022-02-24

**Authors:** Toshihiro Tanaka, Rika Sakai, Ilseung Choi, Junichi Tsukada, Hidenori Sasaki, Yoshiko Naito, Fumiaki Kiyomi, Yasushi Takamatsu, Kazuo Tamura

**Affiliations:** 1 Division of Oncology, Hematology, and Infectious Diseases, Department of Internal Medicine, Fukuoka University, 7-45-1 Nanakuma, Jonan-ku, Fukuoka, 814-0180 Japan; 2 Department of Hematology and Medical Oncology, Kanagawa Cancer Center, Yokohama, 241-0815 Japan; 3 Department of Hematology, National Hospital Organization Kyushu Cancer Center, Fukuoka, 811-1395 Japan; 4 Department of Hematology, University of Occupational and Environmental Health, Kitakyushu, 807-8555 Japan; 5 Statistics and Data Center, Clinical Research Support Center Kyushu, Fukuoka, 812-8582 Japan

**Keywords:** B-cell lymphoma, Oncology, Geriatrics

## Abstract

We conducted a multicenter prospective study on whether a comprehensive geriatric assessment (CGA) can predict the adverse events (AEs) of chemotherapy in elderly patients with diffuse large B-cell lymphoma (DLBCL). Patients aged ≥ 65 years with newly diagnosed DLBCL underwent a pretreatment baseline CGA consisting of six assessment tools: activities of daily living (ADL), instrumental ADL (IADL), mood, nutritional status, comorbidities, and cognitive function. An attending physician chose each patient’s treatment but was blind to CGA results. Patients were grouped as “dependent” or “independent” according to the CGA. The primary endpoint was to evaluate the association between chemotherapy-induced grade 3–4 toxicity and CGA. Of 86 patients, 78 completed the designated CGA. The median age was 79 years (65–89). Seventy-two patients were treated with a cyclophosphamide, doxorubicin, vincristine, and prednisolone (CHOP-like) regimen, and six were treated with low-toxicity regimens. Forty-one patients were classified as dependent and 37 as independent. In multivariate analysis, an impairment of IADL was independently associated with grade 3–4 leukopenia (odds ratio [OR] 0.63; 95% confidence interval [CI] 0.43–0.92, *p* = 0.017) and anemia (OR 0.67; 95% CI 0.50–0.90, *p* = 0.008). The presence of a comorbidity was also associated with grade 3–4 non-hematological toxicity (OR 2.17; 95% CI 1.37–3.43, *p* = 0.001). The 4-year survival rate tended to be longer in the independent (72.7%) compared to dependent (56.9%) group. Overall, a CGA may be a useful tool for predicting serious AEs associated with chemotherapy in elderly patients with DLBCL.

## Introduction

In Japan, the proportion of the population aged ≥ 65 years is increasing and expected to exceed 30% by 2025^1^. Since the prevalence of cancer increases with age, the number of elderly patients with cancer is expected to rise with time. However, evidence for making decisions on appropriate treatments for such elderly patients is lacking. This is because such patients are often excluded from clinical studies owing to a high prevalence of organ dysfunction and other comorbidities, risk of death from other diseases, and incidence of serious adverse events (AEs) in this cohort. Accordingly, treatment decisions for the elderly tend to be based on their chronological age, meaning they may miss out on receiving treatment that would likely improve their quality of life and prognosis.

As a hematological malignancy, diffuse large B cell lymphoma (DLBCL) is a potentially curable disease, even in the elderly, if the appropriate dose intensity of chemotherapy is given. Bosly et al.^2^ treated 210 patients with DLBCL using a CHOP regimen of cyclophosphamide (CPA), doxorubicin (ADR), vincristine (VCR), and prednisolone. Median survival was 7.8, 3.1, and 1.7 years in patients receiving ≥ 90%, 85–90%, and 81–85% of the standard dose, respectively, highlighting how even a slight dose reduction had a significant survival impact. Coiffier et al.^3^ compared full-dose CHOP with R-CHOP (CHOP and rituximab) in elderly with DLBCL. However, several deaths occurred despite reaching a complete response (CR) highlighting the difficulty of treating the elderly.

Performance status (PS) and age have conventionally been employed as reference points to determine chemotherapy regimens and doses as well as to predict AEs. However, since individual differences between elderly patients are quite large, it is necessary to develop an appropriate evaluation tool for predicting the likelihood of AEs. In recent years, a comprehensive geriatric assessment (CGA), based on the concept of a comprehensive evaluation of functions in the elderly, has been useful in geriatrics for drawing up treatment plans and predicting prognoses^4,5^. Specifically, activities of daily living (ADL), instrumental ADL (IADL), cognitive function, and mood/emotion were evaluated^6^. However, which tools to adopt, the preferred combination, and what they are useful for remain to be established in the oncology field. We, thus, retrospectively investigated the association between results of a CGA, consisting of six assessment tools (ADL, IADL, cognitive function, mental status, nutritional status and comorbidities) in the field of geriatrics with prognosis in 98 elderly patients with non-Hodgkin’s lymphoma who received initial treatment between 2004 and 2008. It was found that assessment results correlated with prognosis leading to the conclusion was that these were useful assessment tools in the oncology field^7^. In this prospective, multicenter study (CGAL study), we evaluated the correlation between each CGA domain and AEs resulting from DLBCL therapy as the primary endpoint.

## Patients and methods

### Patients and study design

Eligibility criteria included being aged ≥ 65 years with histologically proven DLBCL. Patient characteristics consisted of age, sex, histological classification, stage, PS, and international prognostic index (IPI)^8^. All patients were evaluated using their medical history, physical examination, imaging and organ function, and a CGA that consisted of ADL, IADL, mood, cognition, nutrition, and comorbidities. Assessments of ADL were based on the Barthel index^9^: patients requiring assistance with at least one of feeding, bathing, walking, dressing, toileting, or grooming were deemed impaired in ADL; similarly, with assessing IADL^10^. Mood was evaluated using the 15-item Geriatric Depression Scale (GDS-15)^11^. A score of ≥ 11 was defined as having a mood disability. Cognitive status was assessed using the revised version of Hasegawa’s dementia scale (HDS-R)^12^; a score of ≤ 20 meant dementia was suspected. Nutrition was assessed based on a mini-nutritional assessment^13^. Of a total of 30 points, 17 points or less were deemed to be problematic. Comorbidities were evaluated using a Charlson comorbidity index^14^ and a score of ≥ 5 was deemed to be problematic. Having one or more problems in six CGA domains was defined as "dependent", whereas the remaining cases were defined as "independent". The primary endpoint of the study was to evaluate the association between grade 3–4 toxicity of treatment and each domain of a CGA. The secondary endpoint was to examine the relationship between the CGA as a whole (dependent or independent) and the following outcomes: total ARDI, treatment intensity (TI), CR rate, causes of death, and overall survival (OS). In addition, as exploratory endpoints, we compared the results of each CGA domain, or CGA as a whole, and baseline clinical factors (PS, age, stage, serum albumin, and lactate dehydrogenase [LDH]) that are considered risks for AEs.

### Treatment and relative chemotherapy dose

Each attending physician, who was blind to CGA results, made decisions on whether to treat a patient, regimen choice, and therapeutic intensity. Treatment with curative intent was based on a combination of standard chemotherapy (CHOP or CHOP-like regimens) with rituximab as shown in Table 1. Patients unable to tolerate such standard treatments received less intensive chemotherapy (mini-CHP [subtraction of vincristine from a 50% dose CHOP], oral etoposide, or sobuzoxane).

**Table 1:** Baseline clinical features of 78 DLBCL patients.

Characteristics	No. of patients (%)
Median age, years (range)	79 (65–89)
65–79	43 (55.1)
≥ 80	35 (44.9)
Sex (male/female)	41/37 (52.6/47.4)
Performance status	
0–1	69 (88.5)
2	8 (10.3)
3	1 (1.3)
4	0 (0.0)
Ann Arbor stage	
I–II	41 (52.6)
III–IV	37 (47.4)
B symptoms	
No	62 (79.5)
Yes	16 (20.5)
International prognostic index	
Low & Low-intermediate	44 (56.4)
High-intermediate & High	34 (43.6)
Treatment regimen	
CHOP like	72 (92.3)
R-CHOP	55 (70.5)
R-CHOP + RTx	7 (9.0)
R-THPCOP	3 (3.8)
R-EPOCH	3 (3.8)
R-ECOP	2 (2.6)
R-CHOEP	1 (1.3)
CHOP	1 (1.3)
Low toxicity regimen	6 (7.7)
R-mini-CHP	2 (2.6)
R-oral sobuzoxane and etoposide	4 (5.1)

*CHOEP* addition of etoposide to CHOP, *CHOP* cyclophosphamide, doxorubicin, vincristine, prednisolone, *mini-CHP* subtraction of vincristine from 50% dose CHOP, *COP* cyclophosphamide, vincristine, prednisolone, *DLBCL* diffuse large B-cell lymphoma, *ECOP* addition of etoposide to COP, *EPOCH* consists of continuously infused etoposide, doxorubicin, cyclophosphamide, vincristine, prednisolone, *R* rituximab, *RTx* radiotherapy, *THPCOP* addition of pirarubicin to COP.

The dose intensities of CPA, ADR, and VCR were based on dose per week (mg/m^2^/week), and the doses actually administered were divided by the respective standard doses to determine their respective relative dose intensities (RDIs). The average RDI (ARDI) was the average actual RDI of each drug (CPA, ADR, and VCR). Total ARDI (%) was the average actual ARDI during the total treatment period. Treatment intensity (%) was calculated by dividing the total actual dose during the total treatment period by the standard total dose in standard courses. For example, six or eight cycles of a standard regimen, without any dose reduction or administration delay for patients with advanced stage, were defined as having a TI value of 100%.

Supportive care for AEs was given according to established guidelines. In principle, granulocyte-colony stimulating factor was used as primary prophylaxis. Sulfamethoxazole-trimethoprim for *pneumocystis pneumonia* prophylaxis was used when rituximab was administered. Similarly, antibiotics at the onset of febrile neutropenia were also administered according to the guidelines.

### Evaluation of toxicity, response, and follow-up

Adverse events were assigned grades based on National Cancer Institute Common Terminology Criteria for Adverse Events version 4.0. Responses were classified as a CR, or partial response (PR), whereas stable disease (SD) and progressive disease (PD) were considered as no response according to International Workshop criteria^15^. The OS was the time interval between the initiation of therapy to death from any cause and evaluated according to the Kaplan–Meier method. Patients were followed up for treatment-related toxicity and survival data from 1 September 2013 to 31 March 2020.

### Statistical analysis

All variables are presented as medians and ranges (min–max) or numbers (%). The characteristics of patient groups classified according to CGA categorization were compared using Pearson’s chi-square test or Fisher’s exact test for nominal scale variables and a Wilcoxon rank sum test for continuous variables. A log-rank test was used to compare actuarial survival curves. The OS rates and 95% confidence intervals (CIs) at four years were estimated based on asymptotic normality using Greenwood's formula. Adverse events were analyzed using simple and multiple logistic regression models with the six assessment tools of a CGA, PS, age, stage, serum albumin, and serum LDH as covariates. A stepwise method was used for variable selection in multiple regression analysis. Statistical analyses were conducted using JMP software (SAS Institute, Cary, NC). *P* < 0.05 was considered statistically significant in all analyses.

### Ethical considerations

We conducted a multicenter, prospective study across four medical institutions (Fukuoka University Hospital, Kanagawa Cancer Center, National Hospital Organization Kyushu Cancer Center, and Hospital of the University of Occupational and Environmental Health) in Japan. This trial was registered with the University Hospital Medical Information Network, Center identifier UMIN (registration number: UMIN000025231). The institutional review board of Fukuoka University [13–8–11] and that of each institution (Kanagawa Cancer Center [26–39], National Hospital Organization Kyushu Cancer Center [2014–13], and Hospital of the University of Occupational and Environmental Health [H26–001]) approved the study protocol. Informed consent was obtained before data collection. Fukuoka University Hospital served as the data-coordinating center for the study and was responsible for entering data on all participants. The study was conducted according to the Declaration of Helsinki and Ordinance on Good Clinical Practice.

## Results

### Patient characteristics and CGA

**Figure. 1:**
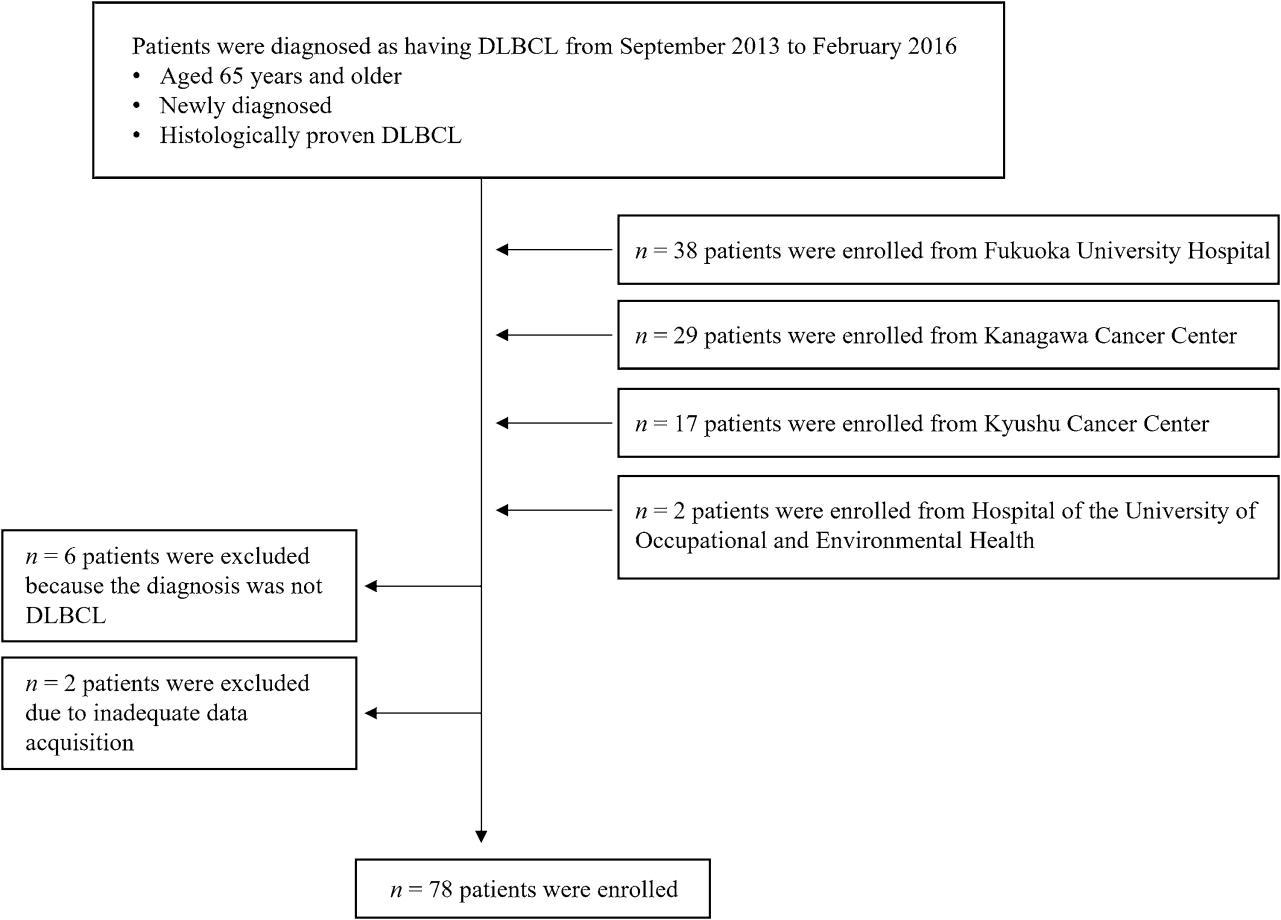
Flow diagram of patients. In four participating institutions, 86 patients aged ≥ 65 years were initially diagnosed with DLBCL from 2013 to 2016. A total of eight patients did not have adequate data, and the remaining 78 patients were enrolled in the present study. *DLBCL,* diffuse large B-cell lymphoma.

From September 2013 to February 2016, 86 patients were consecutively registered; however, only 78 had evaluable data for the present study (Fig. 1). Patient characteristics are summarized in Table 1. The median age was 79 years (range 65–89 years) and 44.9% of patients were aged ≥ 80 years. The PS score was 0 or 1 in 88.5%, 2 in 10.3%, and 3 in 1.3% of patients. Clinical stages were III–IV in 47.4% of patients; the remaining patients had stage I–II disease. Of total patients, 20.5% had B symptoms, and 43.6% belonged to high-intermediate or high-risk categories according to the IPI. A CHOP-like regimen with curative intent was initiated in 92.3% of patients. All but one patient received rituximab.

Table 2 shows the percentage of patients who were found to be vulnerable based on their respective CGA criteria. Limitations in ADL were found in 26.9% of patients, and 14.1% of patients needed assistance in completing IADLs. Nine percent of patients were found to have depression. Cognitive dysfunction was suspected in 10.3% of patients, and nutritional deficits and comorbidities were found in 12.8%, and 15.4%, respectively.

**Table 2:** CGA criteria and vulnerability rates in all patients.

CGA category	Tool of evaluation	Definition	Positive (%)
ADL	Barthel Index	< 100	21 (26.9)
IADL	Lawton and Brody	< 5	11 (14.1)
Psychological status	GDS-15	> 10	7 (9.0)
Cognitive function	HDS-R	≦20	8 (10.3)
Nutritional status	MNA	< 17	10 (12.8)
Comorbidities	CCI	≥ 5	12 (15.4)

*ADL* activity of daily living, *CCI* Charlson comorbidity index, *CGA* comprehensive geriatric assessment, *GDS-15* 15-item geriatric depression scale, *HDS-R* Hasegawa’s dementia scale, *IADL* instrumental activity of daily living, *MNA* mini-nutritional assessment.

According to the CGA, 47.4% of patients were classified as independent and the remaining 52.6% who met at least one of the six frailty criteria were classified as dependent (Table 3). The independent population was significantly younger (*p* < 0.001) and had a good PS (*p* = 0.031) compared to the dependent population, while no significant difference concerning stage and IPI was observed. Regarding the secondary endpoint, no statistically significant difference existed between CGA as a whole (dependent or independent) and the following: total ARDI, TI, and CR rate. Against expectations, the selection rate for less intensive chemotherapy was found to be significantly higher in the independent group (*p* = 0.009); four of the six patients had a PS of 0 and the remaining two had a PS of 1.

**Table 3:** Clinical characteristics of 78 patients classified as dependent or independent according to a CGA.

Variable	Dependent	Independent	*P* value
No. of evaluable patients (%)	41 (52.6)	37 (47.4)	
Male (%)	21 (51.2)	20 (54.1)	NS
Median age (range)	81 (65–89)	72 (65–87)	< 0.001*
PS ≥ 2 (%)	8 (19.5)	1 (2.7)	0.031*
Ann Arbor stage III—IV (%)	21 (51.2)	16 (43.2)	NS
IPI intermediate-high/high (%)	19 (46.3)	15 (40.5)	NS
Less intensive chemotherapy (%)	0 (0.0)	6 (16.2)	0.009*
Total ARDI (%), (range)	74.2 (36.2–100)	82.6 (0.0–100)	NS
TI (%), (range)	50.9 (8.0–100)	79.5 (0.0–100)	NS
CR rate (%)	29 (70.7)	29 (78.4)	NS

*ARDI* average relative dose intensity of cyclophosphamide, doxorubicin, and vincristine, *Total ARDI* average actual ARDI during the total treatment period, *CGA* comprehensive geriatric assessment, *CR* complete response, *IPI* International Prognostic Index, *NS* not significant, *PS* performance status, *P*-value, Wilcoxon rank sum test for continuous variables and Pearson's chi-squared test or Fisher’s exact test for nominal scale variables. *TI* Treatment intensity is the ratio of total actual dose during the total treatment period to the standard total dose in standard courses. *Comparison between dependent and independent.

### Association between treatment toxicity and each CGA domain

Treatment-related deaths did not occur. Overall, 80.8% (63/78) of all patients showed a hematological grade 3–4 toxicity; such patients made up 67.6% (25/37) of the independent group and 92.7% (38/41) of the dependent group (Table 4). The rate of severe hematological toxicity was significantly higher in the dependent than independent group (*p* = 0.013). In particular, the rates of leukopenia and anemia were significantly higher in the dependent group (*p* = 0.013 and 0.020, respectively). Regarding non-hematological toxicities, these were observed in 35.9% of all patients; the incidence was also significantly higher in the dependent compared to independent group (53.7% vs. 16.2%, *p* = 0.001). The frequency of metabolic and nutritional disorders, such as hypoalbuminemia, electrolyte abnormalities, impaired glucose tolerance, and loss of appetite, were significantly higher in the dependent group (*p* < 0.001).

**Table 4:** Treatment-related adverse events (grades 3–4) in patients with DLBCL.

Variable	All patients (n = 78)	Dependent (n = 41)	Independent (n = 37)	*P *value
Hematologic toxicity	63 (80.8)	38 (92.7)	25 (67.6)	0.013*
Leukopenia	53 (67.9)	33 (80.5)	20 (54.1)	0.013*
Neutropenia	60 (76.9)	35 (85.4)	25 (67.6)	NS
Anemia	20 (25.6)	15 (36.6)	5 (13.5)	0.020*
Thrombocytopenia	12 (15.4)	9 (22.0)	3 (8.1)	NS
Febrile neutropenia	20 (25.6)	11 (26.8)	9 (24.3)	NS
Non-hematologic toxicity	28 (35.9)	22 (53.7)	6 (16.2)	0.001*
Metabolic and nutritional disorders	26 (33.3)	22 (53.7)	4 (10.8)	< 0.001*
Weight loss	2 (2.6)	2 (4.9)	0 (0.0)	NS
AST or ALT increased	1 (1.3)	1 (2.4)	0 (0.0)	NS
Gastrointestinal disorders	3 (3.8)	3 (7.3)	0 (0.0)	NS
Cardiac disorders	2 (2.6)	2 (4.9)	0 (0.0)	NS
Infections	6 (7.7)	4 (9.8)	2 (5.4)	NS
Peripheral neuropathy	3 (6.4)	1 (7.3)	2 (5.4)	NS
Cerebral infarction	2 (2.6)	2 (4.9)	0 (0.0)	NS

*AST* aspartate transaminase, *ALT* alanine aminotransferase, *DLBCL* diffuse large B-cell lymphoma, *NS* not significant, *Comparison between dependent and independent; *P* value, Pearson’s chi-squared test or Fisher’s exact test.

As for the primary endpoint, the relationship between each domain of CGA and toxicity is shown in Table 5. In univariate analyses, the following three CGA categories were associated with toxicities of chemotherapy: First, with regard to a correlation between IADL and leukopenia or anemia among patients deemed to have a low IADL at baseline, 100% of such patients experienced severe leukopenia compared with 62.7% of normal scoring patients (*p* = 0.013). Similarly, 54.5% of patients with an IADL disorder experienced severe anemia compared with only 20.9% with a normal score (*p* = 0.025). Next, when examining a correlation between comorbidities and non-hematological toxicities among patients with CCI scores ≥ 5, 83.3% of such patients experienced severe toxicity compared with only 27.3% of those with a score of up to 4 (*p* = 0.002). Third, in correlating cognitive function with leukopenia or anemia among patients with HDS-R ≤ 20, 100% of such patients experienced severe leukopenia compared with 64.3% of normal-scoring patients (*p* = 0.049). Similarly, 62.5% of patients with cognitive dysfunction experienced severe anemia compared with only 21.4% of those with normal function (*p* = 0.021).

**Table 5:** Univariate analyses for CGA domains or clinical factors associated with the occurrence of toxicities (grade 3–4).

Parameter	Category	N	Leukopenia (%)	Neutropenia (%)	Anemia (%)	Thrombocytopenia (%)	FN (%)	Non-hematological (%)
Psychological status	Normal	71	48 (57.6)	54 (76.1)	17 (23.9)	10 (14.1)	17 (23.9)	25 (35.2)
	Depressive	7	5 (71.4)	6 (85.7)	3 (42.9)	2 (28.6)	3 (42.9)	3 (42.9)
	*P value*		0.836	0.569	0.281	0.324	0.286	0.688
	OR (95% CI)		1.20 (0.22–6.65)	1.89 (0.21–16.81)	2.38 (0.48–11.72)	2.44 (0.42–14.34)	2.38 (0.48–11.72)	1.38 (0.29–6.66)
ADL	Normal	57	35 (61.4)	42 (73.7)	14 (24.6)	9 (15.8)	18 (31.6)	18 (31.6)
	Dependent	21	18 (85.7)	18 (85.7)	6 (28.6)	3 (14.3)	2 (9.5)	10 (47.6)
	*P value*		0.051	0.271	0.719	0.87	0.063	0.194
	OR (95% CI)		3.77 (0.99–14.31)	2.14 (0.55–8.32)	1.23 (0.40–3.77)	0.89 (0.22–3.66)	0.23 (0.05–1.09)	1.97 (0.71–5.48)
IADL	Normal	67	42 (62.7)	49 (73.1)	14 (20.9)	10 (14.9)	17 (25.4)	23 (34.3)
	Dependent	11	11 (100.0)	11 (100.0)	6 (54.5)	2 (18.2)	3 (27.3)	5 (45.5)
	*P value*		0.013*	0.059	0.025*	0.782	0.894	0.478
	OR (95% CI)		–	–	4.54 (1.21–17.09)	1.27 (0.24–6.75)	1.10 (0.26–4.64)	1.59 (0.44–5.79)
Nutritional status	Normal	66	43 (65.2)	49 (74.2)	15 (22.7)	9 (13.6)	15 (22.7)	24 (36.4)
	Poor	10	8 (80.0)	9 (90.0)	3 (30.0)	2 (20.0)	3 (30.0)	3 (30.0)
	*P value*		0.361	0.279	0.616	0.597	0.616	0.696
	OR (95% CI)		2.14 (0.42–10.92)	3.12 (0.37–26.48)	1.46 (0.34–6.34)	1.58 (0.29–8.68)	1.46 (0.34–6.34)	0.75 (0.18–3.17)
Comorbidities	Absent	66	45 (68.2)	52 (78.8)	16 (24.2)	10 (15.2)	17 (25.8)	18 (27.3)
	Present	12	8 (66.7)	8 (66.7)	4 (33.3)	2 (16.7)	3 (25.0)	10 (83.3)
	*P value*		0.918	0.364	0.509	0.874	0.956	0.002*
	OR (95% CI)		0.93 (0.25–3.45)	0.54 (0.14–2.05)	1.56 (0.42–5.88)	1.12 (0.21–5.89)	0.96 (0.23–3.97)	13.33 (2.66–66.83)
Cognitive function	Normal	70	45 (64.3)	52 (74.3)	15 (21.4)	10 (14.3)	17 (24.3)	26 (37.1)
	Impaired	8	8 (100.0)	8 (100.0)	5 (62.5)	2 (25.0)	3 (37.5)	2 (25.0)
	*P value*		0.049*	0.187	0.021*	0.434	0.423	0.503
	OR (95% CI)		–	–	6.11 (1.31–28.53)	2.00 (0.35–11.33)	1.87 (0.40–8.66)	0.56 (0.11–3.00)
PS	0–1	69	45 (65.2)	52 (75.3)	18 (26.1)	10 (14.5)	17 (24.6)	23 (33.3)
	≥ 2	9	8 (88.9)	8 (88.9)	2 (22.2)	2 (22.2)	3 (33.3)	5 (55.6)
	*P value*		0.183	0.381	0.803	0.549	0.576	0.202
	OR (95% CI)		4.27 (0.50–36.16)	2.62(0.30–22.44)	0.81 ( 0.15–4.26)	1.69 (0.31–9.31)	1.53 (0.34–6.79)	2.50 (0.61–10.21)
Age	65–79	43	29 (67.4)	33 (76.7)	11 (25.6)	6 (14.0)	12 (27.9)	16 (37.2)
	80≦	35	24 (68.6)	27 (77.1)	9 (25.7)	6 (17.1)	8 (22.9)	12 (34.3)
	*P value*		0.915	0.967	0.989	0.698	0.612	0.789
	OR (95% CI)		1.05 (0.40–2.74)	1.02 (0.35–2.95)	1.01 (0.36–2.80)	1.28 (0.37–4.37)	0.77 (0.27–2.15)	0.88 (0.35–2.24)
Stage	I/II	41	26 (63.4)	30 (73.2)	11 (26.8)	9 (22.0)	11 (26.8)	14 (34.1)
	III/IV	37	27 (73.0)	30 (81.1)	9 (24.3)	3 ( 8.1)	9 (24.3)	14 (37.8)
	*P value*		0.368	0.41	0.8	0.103	0.8	0.734
	OR (95% CI)		1.56 (0.59–4.09)	1.57 (0.54–4.60)	0.88 (0.32–2.43)	0.31 ( 0.08–1.26)	0.88 (0.32–2.43)	1.17 (0.46–2.96)
Serum albumin	< median	39	29 (74.4)	31 (79.5)	12 (30.8)	6 (15.4)	10 (25.6)	16 (41.0)
	> median	39	24 (61.5)	29 (74.4)	8 (20.5)	6 (15.4)	10 (25.6)	12 (30.8)
	*P value*		0.228	0.592	0.302	1	1	0.346
	OR (95% CI)		0.55 (0.21–1.45)	0.75 (0.26–2.16)	0.58 (0.21–1.63)	1.00 (0.29–3.42)	1.00 (0.36–2.76)	0.64 (0.25–1.62)
Serum LDH	< median	39	24 (61.5)	29 (74.4)	7 (17.9)	6 (15.4)	9 (23.1)	12 (30.8)
	> median	39	29 (74.4)	31 (79.5)	13 (33.3)	6 (15.4)	11 (28.2)	16 (41.0)
	*P value*		0.228	0.592	0.124	1	0.604	0.346
	OR (95% CI)		1.81 (0.69–4.76)	1.34 (0.46–3.85)	2.29 (0.80–6.56)	1.00 (0.29–3.42)	1.31 (0.47–3.63)	1.57 (0.62–3.98)
CGA as a whole	Dependent	41	33 (80.5)	35 (85.4)	15 (36.6)	9 (22.0)	11 (26.8)	22 (53.7)
	Independent	37	20 (54.1)	25 (67.6)	5 (13.5)	3 ( 8.1)	9 (24.3)	6 (16.2)
	*P value*		0.015*	0.068	0.024*	0.103	0.8	0.001*
	OR (95% CI)		3.51 (1.28–9.60)	2.80 (0.93–8.46)	3.69 (1.18–11.51)	3.19 (0.79–12.83)	1.14 (0.41–3.16)	5.98 (2.06–17.41)

*ADL* activity of daily living, *CGA* comprehensive geriatric assessment, CI, confidence interval, *FN* febrile neutropenia, *IADL* instrumental ADL, * indicates a significant difference; *PS*, performance status, *LDH*, lactate dehydrogenase, *OR*, odds ratio, *P*-value, Simple logistic regression analysis or Fisher’s exact test if simple logistic regression analysis is not appropriate due to quasi-complete separation.

We also attempted an exploratory univariate analysis of generally suspected risk factors for AEs (PS, age, stage, serum albumin, and LDH). A statistically significant difference between these parameters and severe AEs was not noted. As a result, only the relationships between IADL or cognitive function and hematological toxicity, and between comorbidity and non-hematological toxicity, were found to be statistically significant. Based on these results, we conducted a multivariate analysis of the relationships between AEs and three risk factors (IADL, comorbidity, and cognitive function) as shown in Table 6. Impairment of IADL was independently associated with grade 3–4 leukopenia (odds ratio [OR] 0.63; 95% CI 0.43–0.92) and anemia (OR 0.67; 95% CI 0.50–0.90). Similarly, the presence of a comorbidity was also independently associated with non-hematological toxicities (OR 2.17; 95% CI 1.37–3.43). Further exploratory multivariate analysis that included other risk factors (PS, age, stage, albumin, and LDH) showed that only IADL and comorbidity were statistically significant. Thus, only IADL and the comorbidity index significantly correlated with treatment-related AEs.

**Table 6:** Multivariate analyses of the relationship between CGA and toxicities (grade 3–4).

		Leukopenia	Anemia	Non-hematological
IADL	OR (95% CI)	0.63 (0.43–0.92)	0.67 (0.50–0.90)	
	*P* value	0.017*	0.008*	
Comorbidities	OR (95% CI)			2.17 (1.37–3.43)
	*P* value			0.001*

*CGA* comprehensive geriatric assessment, *CI* confidence interval, *IADL* instrumental activity of daily living, *OR* odds ratio, *indicates significant difference; In multiple logistic regression analysis, raw scores of CGAs without binarization were used as covariates because of quasi-complete separation. Toxicities were analyzed using a multiple logistic regression model including six assessment tools of CGA (performance status, age, Ann-Arbor stage, albumin, and lactate dehydrogenase) as covariates. Variable selection was performed by a stepwise method. Only variables selected as being significantly associated with at least one toxicity in the stepwise method are shown in the table.

### Association between prognosis and CGA as a whole

**Figure 2:**
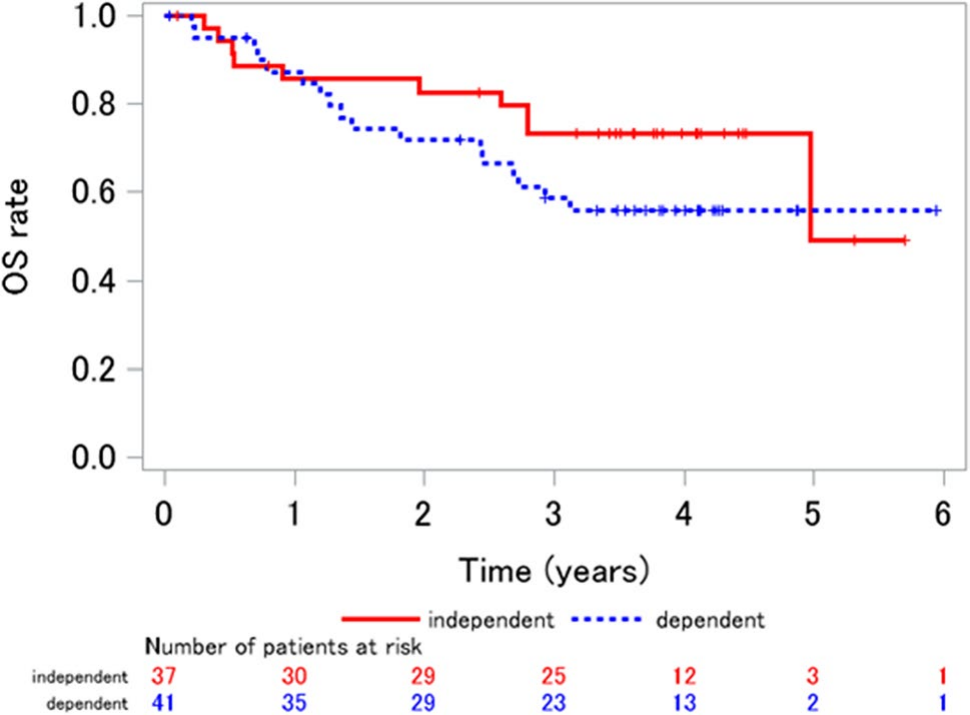
Overall survival curves for DLBCL cases classified as independent and dependent according to a CGA. Red line: independent group (n = 37). Blue line: dependent group (n = 41). The median overall survival (OS) was 4.98 years (95% confidence interval [CI] 2.79–) in the independent group but was not reached in the dependent group (95% CI 1.82–), respectively (log-rank analysis: *p* = 0.180). CGA, Comprehensive Geriatric Assessment; *DLBCL,* diffuse large B-cell lymphoma.

The median observation period was 3.48 years (range 0.036–5.94). Regarding the secondary endpoint, as shown in Fig. 2, the median OS was 4.98 years (95% CI 2.79–) in the independent group while the median OS was not yet reached in the dependent group, respectively (log-rank analysis: *p* = 0.180). The 4-year OS was 72.7% (95% CI 57.4–87.9) among independent patients and 56.9% (95% CI 41.4–72.4) among dependent patients, respectively (*p* = 0.156). Patient deaths made up 27.0% (10/37) of the independent group and 41.5% (17/41) of the dependent group. Lymphoma accounted for 90.0% (9/10) of deaths in the independent group. In comparison, in the dependent group, 47.1% (8/17) of patients died for reasons other than lymphoma, which included pneumonia in three patients; the remaining patients died of heart failure, stroke, gingival cancer, and drowning, at one each, respectively (Table 7).

**Table 7:** Major causes of patient deaths.

Cause of death	Dependent (n = 41)	Independent (n = 37)
No. of deaths (%)	17 (41.5)	10 (27.0)
Lymphoma (%)	8 (47.1)	9 (90.0)
Other than lymphoma (%)	8 (47.1)	1 (10.0)
Pneumonia (%)	3 (17.6)	0 (0.0)
Heart failure (%)	1 (5.9)	0 (0.0)
Renal failure (%)	1 (5.9)	0 (0.0)
Stroke (%)	1 (5.9)	0 (0.0)
Gingival cancer (%)	1 (5.9)	0 (0.0)
Drowning (%)	1 (5.9)	0 (0.0)
Sudden death (%)	0 (0.0)	1 (10.0)
Unknown (%)	1 (5.9)	0 (0.0)

## Discussion

This is the first report of a prospective study on the relationship between CGA and chemo-toxicity in elderly Japanese patients with DLBCL. Primary endpoint multivariate analysis revealed an increased risk of severe leukopenia and anemia in patients with difficulties in at least one IADL item, and an increased risk of non-hematologic toxicity in patients with a Charlson Comorbidity Index score ≥ 5 (Table 6). In comparison, age and PS were not predictors of any AE. As with ADL and IADL, PS is an indicator of physical function. However, in the elderly, IADL, which is composed of more specific assessments, can evaluate abnormalities in physical function in more detail than PS, the latter being a rough assessment; this might explain the prominent association between IADL and AEs. With regard to several large prospective studies, Hurria et al.^16^ assessed PS, blood parameters, and CGA in 500 elderly patients with cancer prior to treatment, and further analyzed those with severe chemo-toxicity. Rather than PS scores, falls within the past 6 months, a limitation in moderate activities, requiring assistance with movement, and limited ability to take oral medications without assistance were identified as significant risk factors. In the present study, vulnerability was divided into “independent” and “dependent” groups using a tool consisting of six domains (Table 2). The median age was significantly lower by 8.5 years in the independent compared to dependent group, with the former having a better PS. However, disease stage and the IPI did not affect CGA, although they usually serve as prognostic factors for DLBCL. These results suggest that the CGA tool employed in the present study adequately assessed the vulnerability of elderly patients.

Gómez et al.^17^ examined clinical parameters (age, PS, B symptoms, bulky lesions, LDH, and IPI) in their exploration of risk factors for treatment-related death in elderly patients with high and intermediate grade non-Hodgkin's lymphoma and claimed that only PS was a predictor. In a recent report, Oiwa et al.^18^ used a simple geriatric screening tool (G8) for functional assessment with the addition of IPI, age, PS, serum LDH, stage, number of extra-nodal lesions, serum albumin, bulky lesions, and total ARDI. As a result, they claimed that only bulky lesions and G8 scores, not age or PS, were predictors of treatment-related AEs. As exploratory endpoints, we added CGA parameters, age, and PS, as well as serum albumin level, serum LDH, and stage to the univariate analysis. The IADL and cognitive function were found to be associated with hematological toxicity and the comorbidity index with non-hematological toxicity, but none of the additional parameters examined had influenced AEs.

In this study, notably, all six patients who received less intensive chemotherapy, even though they had no problems with a CGA (independent group), includes the following: Four of the six patients had a PS of 0, and the other two had a PS of 1. All of the patients were over 80 years of age and from the same site. Each attending physician selected treatment without knowing the results of the CGA that suggests that they decided on a treatment plan based on age alone. This highlights a discrepancy between the clinical judgment of the attending physician and the CGA-based determination of the degree of vulnerability in elderly patients with DLBCL. Furthermore, this infers that differences existed in treatment policies between institutions or doctors for such patients.

With regard to a secondary endpoint, the final TI tended to be lower in the dependent (50.9%) compared to independent (79.5%) group. About half of patients in the dependent group were forced to reduce dose intensity due to AEs from chemotherapy. The total ARDI did not differ between both groups, but toxicities were significantly higher in the dependent compared to independent group. Additionally, chemotherapy tended to be discontinued in the dependent group. Of 78 patients, 15 (19.2%) failed to complete their planned chemotherapy (nine [22.0%] dependent; six [16.2%] independent). Of these 15 patients, five discontinued therapy due to disease progression. The outcomes for the remaining 10 (seven dependent, three independent) were five deaths from a cause other than lymphoma (four dependent, one independent) while five remained alive; the average TI was 64.8%. Interestingly, examination of the causes of death showed that 41.5% of the dependent group died during the observation period, with only eight deaths (47.1%) due to malignant lymphoma as the primary disease. In comparison, 27.0% of patients died in the independent group, with nine cases (90.0%) due to lymphoma (Table 7). The rate of non–tumor-related mortality was higher in the dependent (52.9%) than independent (10.0%) group, suggesting that a decline in physical function and the presence of comorbidities may have been responsible for deaths in the former. In other words, the dependent group may have been overtreated, whereas a proportion of the independent group may have been undertreated. We evaluated the therapeutic intensity of chemotherapy with total ARDI (average actual ARDI during the total treatment period) and TI (ratio of total actual dose to the standard total dose in standard courses). Non-elderly patients who can participate in randomized controlled trials or clinical trials are less likely to deviate significantly from standard courses, so total ARDI is likely to almost adequately represent therapeutic intensity. In comparison, elderly patients are often forced to discontinue treatment due to an AE, even if chemotherapy is started at standard doses, resulting in a difference between total ARDI and TI. Differences also exist between total ARDI and TI when comparing patients who were able to receive a near-standard treatment and those who prematurely discontinued treatment, even in the same elderly cohort. The CR rate did not differ significantly between independent (78.4%) and dependent (70.7%) groups. However, the four-year survival rate (72.7% vs. 56.9%) tended to be higher in the independent group. Tucci et al*.*^19^ divided DLBCL patients aged ≥ 65 years into fit and unfit groups based on a simple geriatric assessment incorporating age, ADL, comorbidities, and geriatric syndrome. As in our study, the physician in charge of each patient was not informed of the fit/unfit classification before choosing a therapeutic approach at their discretion, and treatment outcome was examined afterward. In the unfit group, the OS curve was comparable between those receiving intensive chemotherapy or palliative therapy. However, it should be noted that this data is an analysis of a small number of cases from a single institution and that the PS and empirical judgment of the physician in charge alone do not lead to optimal treatment decisions. Elderly populations are characterized by large individual differences in fitness, even though all are physiologically aging. In other words, quite a few elderly patients were treated with a standard dose normally used for younger patients (i.e., classified as fit). Therefore, it is important to identify difficult-to-treat patients, with those prone to developing serious AEs more likely to be unfit or frail. Accordingly, consideration should be given to reducing the dose, prolonging the washout period of the next treatment, and possibly avoiding aggressive treatment in such patients. However, some cases in the dependent group have now survived for 4 years with a TI of around 50%; this might be dependent on the individual biological properties of DLBCL. Therefore, it seems that the treatment plan for elderly with DLBCL should not be decided using CGA alone.

The likelihood of withdrawal from treatment as a result of an AE is higher in elderly patients receiving chemotherapy, and the use of a CGA may aid in predicting toxicity in such patients. American Society of Clinical Oncology guidelines suggest using either a Cancer and Aging Research Group or Chemotherapy Risk Assessment Score for High-Age Patients^20^ tool to obtain specific estimates on the risk of chemotherapy-related toxicities^21^. Japanese guidelines^22^ for elderly cancer patients raised the clinical question: “Is geriatric assessment useful in determining a treatment strategy for DLBCL?”. Guidelines recommend that geriatric assessment should not be used to determine a treatment strategy owing to the lack of a high-quality study. However, CGA is proposed as a tool for detecting overlooked physical and psychological difficulties, whilst acknowledging and understanding a patient’s condition.

We believe that our multicenter, prospective study showed that chronological age and PS, at least, cannot predict treatment-related AEs in elderly patients with DLBCL. We found that patients with an IADL impairment or high comorbidity index need to be carefully monitored for AEs. Since DLBCL is a disease that is highly sensitive to chemotherapy and can be successfully treated by chemotherapy that is as close to standard as possible, it is important to select the appropriate indication for treatment even in the elderly. However, data from a study of the association between CGA and AEs is not useful for selecting cases for standard treatment. Because of the variety and complexity of factors related to vulnerability in the elderly, it is not feasible to conduct a therapeutic intervention study based on CGA alone. A significant trade-off exists between an increase in lymphoma deaths and a decrease in treatment-related AEs or mortality rates of diseases other than lymphoma when reducing the intensity of chemotherapy along with CGA results. Further study is needed to confirm a CGA as a prognostic factor that can become a decision-making tool for choosing an appropriate treatment strategy in elderly patients with DLBCL.

## Data Availability

Study data are available from the corresponding author on reasonable request.

## References

[1] The Editorial Board of the Cancer Statistics in Japan. Cancer Statistics in Japan’19. Foundation for promotion of Cancer Research. https://ganjoho.jp/data/reg_stat/statistics/brochure/2019/cancer_statistics_2019.pdf (2020).

[2] Bosly A (2008). Achievement of optimal average relative dose intensity and correlation with survival in diffuse large B-cell lymphoma patients treated with CHOP. Ann. Hematol..

[3] Coiffier B (2002). CHOP chemotherapy plus rituximab compared with CHOP alone in elderly patients with diffuse large-B-cell lymphoma. N. Engl. J. Med..

[4] Wildiers H (2014). International society of geriatric oncology consensus on geriatric assessment in older patients with cancer. J. Clin. Oncol..

[5] Extermann M (2005). Use of comprehensive geriatric assessment in older cancer patients: recommendations from the task force on CGA of the International Society of Geriatric Oncology (SIOG). Crit. Rev. Oncol. Hematol..

[6] Stuck AE, Siu AL, Wieland GD, Adams J, Rubenstein LZ (1993). Comprehensive geriatric assessment: a meta-analysis of controlled trials. Lancet.

[7] Naito Y, Sasaki H, Takamatsu Y, Kiyomi F, Tamura K (2016). Retrospective analysis of treatment outcomes and geriatric assessment in elderly malignant lymphoma patients. J. Clin. Exp. Hematop..

[8] The International Non-Hodgkin’s Lymphoma Prognostic Factors Project (1993). A predictive model for aggressive non-Hodgkin’s lymphoma. N. Engl. J. Med..

[9] Mahoney FI, Barthel DW (1965). Functional evaluation: The Barthel Index. Md. State Med. J..

[10] Lawton MP, Brody EM (1969). Assessment of older people: self-maintaining and instrumental activities of daily living. Gerontologist.

[11] Yesavage, J. A. *et al*. Development and validation of a geriatric depression screening scale: a preliminary report. *J. Psychiatr. Res.* **17**, 37–49 (1982–1983). 10.1016/0022-3956(82)90033-4 7183759

[12] Jeong JW (2007). A normative study of the Revised Hasegawa Dementia Scale: comparison of demographic influences between the Revised Hasegawa Dementia Scale and the Mini-Mental Status Examination. Dement. Geriatr. Cogn. Disord..

[13] Guigoz, Y, Lauque, S., Vellas, B. J. Identifying the elderly at risk for malnutrition. The Mini Nutritional Assessment. *Clin. Geriatr. Med.* **18**, 737–757 (2002). 10.1016/s0749-0690(02)00059-9 12608501

[14] Charlson ME, Pompei P, Ales KL, MacKenzie CR (1987). A new method of classifying prognostic comorbidity in longitudinal studies: development and validation. J. Chronic Dis..

[15] Cheson B. D. *et al.* Report of an international workshop to standardize response criteria for non-Hodgkin’s lymphomas. NCI Sponsored International Working Group. *J. Clin. Oncol.* **17**, 1244. (1999). Erratum in: *J. Clin. Oncol.* **18**, 2351 (2000). 10.1200/JCO.1999.17.4.1244 10561185

[16] Hurria A (2011). Predicting chemotherapy toxicity in older adults with cancer: A prospective multicenter study. J. Clin. Oncol..

[17] Gómez H (1998). Risk factors for treatment-related death in elderly patients with aggressive non-Hodgkin's lymphoma: results of a multivariate analysis. J. Clin. Oncol..

[18] Oiwa K (2021). Utility of the geriatric 8 for the prediction of therapy-related toxicity in older adults with diffuse large B-cell lymphoma. Oncologist..

[19] Tucci A (2009). A comprehensive geriatric assessment is more effective than clinical judgment to identify elderly diffuse large cell lymphoma patients who benefit from aggressive therapy. Cancer.

[20] Extermann M (2012). Predicting the risk of chemotherapy toxicity in older patients: The Chemotherapy Risk Assessment Scale for High-Age patients (CRASH) score. Cancer.

[21] Mohile SG (2018). Practical assessment and management of vulnerabilities in older patients receiving chemotherapy: ASCO guideline for geriatric oncology. J. Clin. Oncol..

[22] Japanese Society of Medical Oncology, Japan Society of Clinical Oncology. Chemotherapy and other drug therapies for older patients with cancer: JSMO-JSCO clinical practice guidelines. Available via https://www.jsmo.or.jp/about/doc/guideline01_03.pdf (2019).

